# *De novo* assembly and analysis of changes in the protein-coding transcriptome of the freshwater shrimp *Paratya australiensis* (Decapoda: Atyidae) in response to acid sulfate drainage water

**DOI:** 10.1186/s12864-016-3208-y

**Published:** 2016-11-07

**Authors:** Peter A. Bain, Adrienne L. Gregg, Anupama Kumar

**Affiliations:** 1 Commonwealth Scientific and Industrial Research Organisation, Waite Road, Urrbrae, 5064 Australia; 2 Commonwealth Scientific and Industrial Research Organisation, Private Mail Bag 2, Glen Osmond, 5064 Australia

**Keywords:** Decapod crustaceans, Aquatic toxicology, Transcriptomics, Acid sulfate soils, Toxicogenomics

## Abstract

**Background:**

The atyid shrimp *Paratya australiensis* occurs in surface freshwater habitats throughout eastern Australia and has been used to study the ecotoxicology of contaminants such as pesticides and metals. The acidification of surface water that can occur after acid sulfate material in soils and sediments is oxidised and subsequently re-wetted is a serious environmental issue in coastal regions and inland riverine floodplains worldwide. Solubilisation of soil-associated minerals can result in high waterborne concentrations of mineral salts and dissolved metals, which together with low pH represent a potential threat to aquatic ecosystems in affected regions. The aims of the present study were to gain insight into stress responses induced by exposure to acid drainage water (ADW) in *P. australiensis* by determining changes in the abundance of protein-coding transcripts and to generate a comprehensive transcriptomic resource to facilitate further research into gene regulation or protein structure and function in this species. Adult *P. australiensis* were exposed for 24 h to undiluted ADW, 50 % ADW diluted in river water, or to river water as control, and high-throughput mRNA sequencing (RNA-Seq) conducted on whole-body tissues. A reference transcriptome was generated using *de novo* assembly and putative protein-coding regions were identified and annotated. Changes in transcript abundance in response to ADW exposure were determined by aligning reads to the reference transcriptome and quantifying coverage.

**Results:**

A high proportion of arthropod benchmarking universal single-copy orthologues were present in the reference transcriptome. Functions associated with cuticle biosynthesis and oxidative stress were significantly enriched in the lists of transcripts exhibiting differential abundance in either direction after exposure to 50 % or 100 % ADW. Transcripts involved in osmoregulation exhibited decreased abundance following exposure to ADW. The transcriptome contained full-length coding sequences for numerous proteins known to be involved in environmental response pathways, including two putative metallothioneins, four glutathione peroxidases and 19 nuclear receptors.

**Conclusions:**

The results of the present study provide insight into stress response pathways induced in crustaceans by short-term exposure to multiple stressors present in ADW such as low pH, high salinity and dissolved metals, and represent a resource for future toxicogenomics and protein functional studies in *P. australiensis*.

**Electronic supplementary material:**

The online version of this article (doi:10.1186/s12864-016-3208-y) contains supplementary material, which is available to authorized users.

## Background

*Paratya australiensis* (Kemp, 1917) is an atyid shrimp that plays an important role in surface freshwater ecosystems throughout eastern Australia, controlling periphyton biomass through grazing [1] and providing prey for fishes [2, 3]. Because of its small size, short maturation time, and availability through commercial aquarium suppliers, *P. australiensis* is often used to study the aquatic ecotoxicology of environmental contaminants such as pesticides [4–9] and metals [10, 11].

Potential acid sulfate soils and sediments, characterised by high amounts of sulfides present primarily as iron sulfide (pyrite), occur in coastal areas, inland lakes and floodplains throughout the world (reviewed in [12]). Desiccation due to extended drought or land use changes can result in the exposure of sulfides to air and oxidation to sulfuric acid. Following rainfall or the resumption of irrigation, highly acidic runoff can dissolve soil-associated minerals, resulting in high concentrations of salts and metals entering surface water bodies via drainage channels [13–17].

Recently, thousands of hectares of reclaimed floodplains in the Lower Murray region of southern Australia were subjected to an extended period of drying as a result of hydrological changes and reduced irrigation associated with a prolonged drought. This was followed by a series of heavy rainfall events that flushed large volumes of highly acidic water containing metals and salts into drainage channels that discharge into the Murray River [18]. The effects of exposure to acid drainage water (ADW) in organisms within the immediate receiving environment of such discharges are currently not well understood. Crustaceans may particularly susceptible to ADW due to the potential for solubilisation of minerals important for cuticle strength during exposure to low pH conditions [19]. Because of the presence of multiple potential stressors (low pH, high ionic strength and high dissolved metal concentrations), determining the response pathways induced by exposure to ADW may provide insight into mechanisms of adaptation in crustaceans in response to diverse environmental challenges.

The use of high-throughput toxicogenomics methodology is increasingly being employed by researchers to help decipher the effects of complex environmental stressors. Next-generation sequencing of complementary DNA (cDNA) provides a means of unbiased analysis of changes in the protein-coding transcriptome, with the advantage of allowing estimation of read coverage across the entire length of mRNA (including putative splicing variants) rather than targeting regions common to multiple splice variants using oligonucleotide probes typically used in current microarray technologies. *De novo* assembly of sequence reads derived from mRNA also provides full-length protein coding information that can be used to establish phylogenetic relationships and facilitate detailed functional studies of stress-responsive proteins in organisms for which genome sequence data is not available [20].

In the present study, adult *P. australiensis* were exposed to diluted and undiluted ADW containing a range of metals ions and dissolved solids, and toxicogenomic responses determined using RNA-Seq, *de novo* transcriptome assembly, and transcript abundance estimation via read mapping. The goals of the study were to gain insight into gene expression networks involved in responses to ADW exposure in crustaceans and to generate a high-quality transcriptome representing a large proportion of the protein-coding genes to facilitate future ecotoxicogenomics and protein functional studies in *P. australiensis*. Differential transcript abundance in response to ADW was determined and coding sequences for environmental response proteins were identified and analysed.

## Methods

### Animals and exposures

Adult *P. australiensis* were purchased from Aquarium Industries (Victoria, Australia) and maintained at 22 °C in modified FETAX solution [21] under a 16 h:8 h light:dark photoperiod. Groups of 10 adult *P. australiensis* (>13 mm in length) were exposed in triplicate for 24 h at 22 °C to 100 % ADW, 50 % ADW in Murray River water, or 100 % Murray River water as control. After 24 h, shrimp were removed from exposure waters, placed into 2 mL polypropylene tubes, snap-frozen in liquid nitrogen and stored at -80 °C.

### Next-generation sequencing of mRNA (RNA-Seq)

Three individuals (one individual from each of triplicate exposure vessels) were selected at random from each experimental condition. Animals were not sexed prior to RNA extraction. Total RNA was isolated from the whole body omitting the 4-6^th^ abdominal segments and tail fin in order to ensure that the maximum binding capacity of the RNA extraction column was not exceeded. The RNeasy Lipid Kit (QIAGEN) was used to isolate total RNA according to the manufacturer’s protocol. Tissues were homogenised using a FastPrep homogeniser (MP Biomedicals). RNA purity was confirmed spectrophotometrically using a NanoDrop 1000 (NanoDrop Products) and RNA integrity was determined using an Agilent 2100 Bioanalyzer (Agilent Technologies) using the RNA Nano chip. 10 μg of total RNA was treated with RNase-free DNase (Turbo DNA-Free kit; Thermo Fisher Scientific) to remove contaminating genomic DNA.

Strand-specific sequencing of poly-A^+^ mRNA was contracted to an external service provider (Australian Genome Research Facility, Melbourne, Australia). A single cDNA library was prepared for each individual using the TruSeq Stranded mRNA Library Prep kit (Illumina Inc.) and sequenced using the HiSeq2000 instrument (Illumina Inc.) in 100-cycle paired-end mode.

### *De novo* transcriptome assembly

Overall read quality statistics were determined with the aid of FastQC v. 0.10.1 [22]. Quality-based read trimming was carried out using Trimmomatic v. 0.32 in paired-end mode [23, 24]. Due to the base composition bias in the initial portion of the reads, which is thought to be attributable to non-random base distributions in synthetic oligonucleotides used for priming cDNA synthesis [25], the first 14 bases were removed from all reads in all libraries as previously described [26]. Bases with Phred quality scores of below 10 were trimmed successively from the 3’ ends of each read.

*De novo* assembly of putative transcripts was carried out using the Trinity software package [27, 28] version 2.0.6. We implemented an option denoting requirement for a minimum coverage of two identical kmers for contig extension to occur. Assembly statistics were computed using scripts included with the Trinity software package.

The completeness of the transcriptome assembly was estimated by identifying the presence of deduced proteins homologous to a set of benchmarking universal single-copy orthologues (BUSCOs) present in the majority of arthropods, with the aid of BUSCO software [29], and by comparison of the full list of *P. australiensis* deduced proteins to proteomes derived from three currently available arthropod genome sequences – water flea (*Daphnia pulex*), fruit fly (*Drosophila melanogaster*) and termite (*Zootermopsis nevadensis*). The latter analysis was achieved using the BLAST + software suite [30] as follows. A searchable database was generated from the entire list of deduced *P. australiensis* proteins using the makeblastdb program. The full list of predicted proteins from *D. pulex* was obtained from the Joint Genome Institute Genomes OnLine Database (GOLD) [31] and used to query the full set of *P. australiensis* deduced proteins for highly similar sequences using the blastp program. Results were limited to the best hit for each query by selecting output format 6 and specifying a single target sequence be returned (the search parameters used were -evalue 1.0e-5 -num_threads 8 -max_target_seqs 1 -outfmt ‘6 qseqid sseqid stitle pident length mismatch gapopen qstart qend sstart send evalue bitscore qcovs’). For some query sequences, more than one alignment was returned for a single target sequence; the unique alignments were retained using standard linux text processing tools. The same procedure was performed for the predicted proteomes of *D. melanogaster* [32] and *Z. nevadensis* [33].

### Identification of likely protein-coding transcripts

Candidate open reading frames (ORFs) of a minimum length of 300 bp were identified using Transdecoder v. 2.0.1 [27, 34]. Deduced protein sequences were clustered using CD-HIT v. 4.6.4 [35] to generate a subset of non-redundant protein sequences with less 95 % than amino acid identity. The longest sequence from each cluster was retained for downstream analyses.

### Confirmation of species identity

To confirm the identity of the species sequenced in the present study, a sequence homologous to mitochondrial cytochrome c oxidase subunit I (COI) was identified in the transcriptome assembly using reciprocal sequence similarity searches as follows. A *P. australiensis* COI cDNA sequence derived from the complete mitochondrial genome (GenBank accession KM978917) was used to search the *P. australienesis* transcriptome generated in the present study. Two transcripts, TR30545|c0_g1_i1 and TR18069|c0_g3_i1, contained regions that matched the *P. australiensis* COI (GenBank accession KM978917) with 95.5 % nucleotide identity over the full coding region of 1 542 bases. These transcripts were then used as queries to search the entire non-redundant GenBank nucleotide database for similar sequences, which confirmed that the best hit for both transcripts was the *P. australiensis* COI sequence used as the original search query. Because of the relatively high COI sequence diversity among *P. australiensis* sub-populations [36], the nucleotide sequence identity value obtained (95.5 %) can be considered as sufficiently high to confirm the species identity of the animals sampled in the present study. The next-best species of origin was the atyid shrimp *Neocardinia denticulata*, with a match of approximately 85 % with the COI sequence from the complete mitochondrial genome (GenBank accession JX156333).

### Functional annotation of deduced proteins

To identify likely homologues of proteins with known function, sequence similarity searches were performed against the NCBI GenBank non-redundant (nr), UniProtKB-Trembl, and UniprotKB-SwissProt protein databases using in-house batch search tools (CSIRO Bioinformatics Core), using an e-value cutoff of 1e-5. Functional terms provided by the Gene Ontology (GO) consortium [37] were assigned to deduced protein sequences using Blast2GO command-line v. 1.0.2 [38] via an in-house web application (CSIRO Bioinformatics Core). NCBI conserved domain (CD) searches [39] were performed using reverse position-specific BLAST as implemented in the NCBI Batch CD Search [40] to infer additional functional terms by identifying conserved functional or structural domains within the *P. australiensis* deduced protein sequences. Geneious v8 [41] was used to prepare multiple sequence alignments, as a front-end for sequence similarity searches and phylogenetic analyses and for general curation and review of sequence data. Query coverage histograms were prepared in R using the built-in Stats package [42].

### Transcript abundance estimation and functional enrichment analysis

Paired read libraries (i.e. omitting reads that were unpaired after quality-based read trimming) were aligned to transcripts corresponding to non-redundant deduced protein sequences (<95 % identity using CD-HIT) using bowtie2 v. 2.2.4 [43]. Bowtie2 options used for the alignments were ‘-k 200 -X 600 --rdg 6,5 --rfg 6,5 --score-min L,-.6,-.4 --no-discordant --no-mixed -p 10’. Transcript abundance was estimated with eXpress v.1.5.1 using the default parameters for stranded libraries [44]. To omit lowly expressed transcripts from the analysis, the dataset was pre-filtered to retain only transcripts with read counts of greater than an arbitrary cutoff of 100 in any one library. The R package DESeq2 [45] was used to determine differential transcript abundance among the treatments. Differences in transcript abundance were considered to be biologically relevant when changes relative to controls were greater than 2-fold in either direction and *P* values adjusted using Benjamini-Hochberg multiple testing correction (abbreviated herein as *P*_*adj*_) were less than 0.05. Relative transcript abundance datasets were visualised with the aid of CanvasXpress [46] and the R package gplots [47].

Enrichment of functional terms associated with lists of upregulated or downregulated transcripts was determined using Fisher’s exact test as implemented in Blast2GO v. 3.2.7. The Fisher’s exact test false discovery rate (FDR) was considered statistically significant at FDR < 0.05.

### Phylogenetic analyses

Multiple sequence alignments were prepared with the aid of Geneious v8.0.5 [41]. Phylogenetic relationships were inferred by either neighbour-joining [48] using the Geneious tree builder, or maximum likelihood [49, 50] using the PhyML plugin in Geneious, as indicated in the text.

### Quantitative reverse-transcriptase PCR

Changes in the abundance of selected transcripts in response to ADW were validated in the same samples used for RNA-Seq analysis using quantitative reverse transcriptase PCR (qRT-PCR). Primers are presented in Additional file 1: Table S1. Briefly, after DNase treatment (Turbo DNA-free; Life Technologies, Inc., Australia) to remove genomic DNA, total RNA was quantified using the Quanti-iT RiboGreen RNA Assay kit (Invitrogen Life Technologies). Complementary DNA (cDNA) was synthesised from 1 μg of total RNA using the QuantiTect Reverse Transcription kit (Qiagen). Quantitative PCR (qPCR) reactions contained 1 × Brilliant III Ultra-Fast SYBR Green qPCR master mix (Agilent Technologies), 0.2 μM each primer (Geneworks) and 2 μL 10-fold diluted cDNA, in a final volume of 20 μL. 40-cycle qPCR reactions were run using the AriaMx Real-Time PCR System (Agilent Technologies). The amplification of a single product for each primer set was confirmed by the visual inspection of dissociation curves measured between 72 °C and 95 °C. Cycle thresholds (Ct) were determined using the AriaMx integrated software.

Messenger RNA levels for genes of interest were calculated as ratios relative to solvent controls using the 2^-ΔΔC*T*^ method [51], with ribosomal protein S7 as the reference gene. The PCR efficiency for all primer pairs was found to be close to 100 % during primer validation experiments, and was assumed to be 100 % for calculating relative mRNA levels. Abundance ratios relative to solvent controls were log_2_-transformed prior to performing one-way ANOVA followed by Dunnett’s test as implemented in Prism v.6 (GraphPad Inc., CA, USA). Differences in mRNA levels between ADW-exposed and control groups were considered significant at a multiply-adjusted *P*-value (*P*_*adj*_) of less than 0.05.

## Results and discussion

### Laboratory exposures

Water quality parameters and total dissolved metal concentrations for undiluted ADW and river water are available in Additional file 1: Table S2. ADW contained high concentrations of dissolved salts (electrical conductivity of 36.5 mS/cm) and metals (e.g. 55.8 mg/L Fe, 13 mg/L Mn, 1.4 mg/L Al, and 465 μg/L Zn) and was highly acidic (pH 3.17). Basic water quality parameters (pH, dissolved oxygen and electrical conductivity [EC]) for the laboratory exposures are available in Additional file 1: Table S3. The pH during the exposures was 4.17 and 3.05 in 50 % and 100 % ADW, respectively, compared with the river water control pH of 7.55. Despite these challenging conditions, no mortality was observed among the treatments at the end of the exposure period.

### RNA-Seq and *de novo* transcriptome assembly

Mature mRNA from nine individuals (three from each exposure condition) was sequenced on a single lane of the Illumina HiSeq 2000 platform in 100-cycle paired-end mode. The number of paired-end reads obtained for each sample is available in Additional file 1: Table S4. Read quality metrics indicated that all libraries returned mean Phred scores of above 28 for the entire read length, indicating high overall read quality. After trimming of individual reads based on quality, a total of 383,541,818 reads were used as input for *de novo* transcript assembly. A total of 187,435 transcripts were assembled from 25-base kmers comprising 172,384,778 bases. Statistics relevant to overall assembly quality are summarised in Table 1. The contig N50 statistic denotes the length of transcripts above which 50 % of the total assembled nucleotides are included, while the E90N50 statistic denotes the N50 for a subset of sequences representing the top 90 % abundant transcripts in terms of normalised read coverage. For the present study, 30,930 sequences (of 187,435 assembled transcripts) were included this top-90 % subset, indicating that a large proportion of the total number of assembled sequences were expressed at relatively low levels.

**Table 1:** Statistics of the transcriptome assembly

Total assembled bases	172,384,778
Total trinity transcripts	187,435
Total trinity ‘genes’	132,799
Median contig length	415
Mean contig length	920
Contig N50	1884
Contig E90N50	2864

**Fig. 1:**
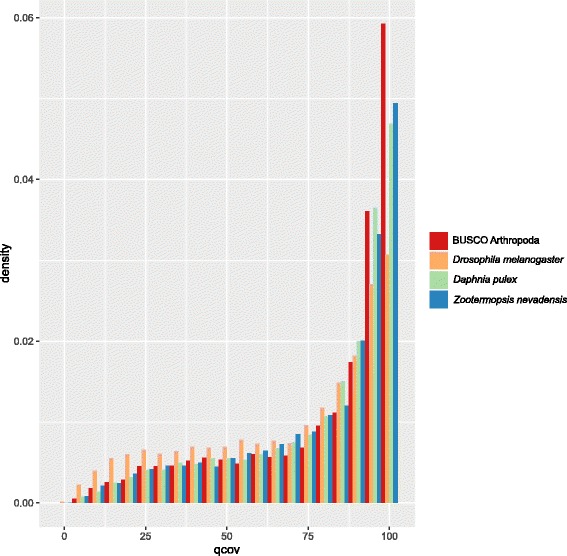
Estimating the completeness of the P. australiensis transcriptome assembly by examining the length of alignments of deduced proteins with arthropod BUSCO consensus sequences or homologous proteins predicted from complete genome sequences of other members of the Pancrustacea clade. Distributions represent alignment length as a percentage of query length (query coverage [qcov], with 5 % bin size) resulting from blastp searches using an e-value cutoff of 1e-5

We did not expect to obtain transcripts representing all protein-coding genes from the *P. australiensis* genome in the present study, which was limited to adults of unknown reproductive status exposed to a limited set of environmental conditions. Therefore, we were interested in estimating the degree of genomic coverage, or completeness, that the assembled transcriptome represented. Determining the presence of benchmarking sets of universal single-copy orthologues (BUSCOs) present a transcriptome assembly can provide a good estimate of completeness in terms of breadth of coverage [29]. The completeness of the assembly presented here was found to be adequate given that only adults were sequenced, with 2295 of 2675 arthropod BUSCOs (85 %) present in the non-redundant set of *P. australiensis* deduced protein sequences. Identifying homologues of proteins derived from sequenced genomes of related species can also provide an approximation of the completeness of a transcriptome assembly in terms of both the number of homologues present as well as their relative length in order to establish the proportion of likely full-length coding sequences. We have used this approach previously for a teleost fish transcriptome analysis [26]. Because of the current lack of availability of a draft genome assembly for any decapod crustacean, in the present study we used proteomes derived from the sequenced genomes of dampwood termite (*Zootermopsis nevadensis*), water flea (*Daphnia pulex*) and fruit fly (*Drosophila melanogaster*), in addition to arthropod BUSCO consensus sequences [29]. Alignment length distributions for significant hits are shown in Fig. 1. For *Z. nevadensis*, 9945 proteins deduced from the *P. australiensis* transcriptome assembly returned significant hits from a total 14,610 *Z. nevadensis* queries (68.1 %). *P. australiensis* homologues were found for less than half of the total number of *D. pulex* proteins (15,211 of 30,810 proteins, or 49.4 %). The number of homologues found for *D. melanogaster* proteins was highest, at 23,600 of 30,362 queries (77.7 %). However, the proportion of full-length hits found for *D. melanogaster* was lower than that found for other species, possibly as a result of more distant overall sequence identity for *D. melanogaster* proteins, as could be expected based on relative taxonomic distances. The current lack of availability of a complete decapod genome presented a challenge for this approach, and it is likely that a greater proportion of homologues would have been found for a more closely related organism. Nonetheless, the presence in the *P. australiensis* transcriptome of a large number of full-length or near full-length coding sequences is demonstrated.

### Confirmation of species identity

A mitochondrial COI sequence was identified in the *P. australiensis* transcriptome and used to confirm the identity of the species used in the present study. By performing a simple phylogenetic analysis of all publicly available sequence data representing *P. australiensis* COI variants (Additional file 2), we identified isolates from a study by Cook et al. [36] that were most similar to the *P. australiensis* strain obtained for the present study. These were found to be isolates SHC32 (GenBank AY308120), H15 (AY308089) and haplotype 82 (AY641780), corresponding to *lineage 6*, which occurs in a wide geographical area on the east coast of Australia and inland river systems bounded by the Goulburn River in Victoria and the Condamine River in Queensland [36].

### Identification and functional annotation of protein-coding transcripts

Likely protein-coding regions within transcripts were identified using a Markov model-based approach [27], which returned a total of 54,302 candidate open reading frames (ORFs) with a minimum length of 300 bp. Of these, 26,347 could be classed as complete in that they encompassed an entire ORF including a start codon and a stop codon, while the remainder were missing start or stop codons and therefore represent partial ORFs.

A considerable degree of redundancy can expected to be present in any protein-coding transcriptome because of the presence of single-nucleotide polymorphisms (both inter-individual and allelic), due to intrinsic mechanisms such as alternative mRNA splicing, and as a result of sequencing or assembly errors. This redundancy can interfere with differential transcript abundance analyses that rely on the use of *de novo* transcriptome assemblies as reference sequences [52]. To reduce redundancy in the dataset, protein sequences with greater than 95 % identity were clustered and the longest sequence from each cluster selected as a representative sequence. This resulted in a list of 32,884 non-redundant protein sequences including those derived from partial ORFs. Subsequent analyses including functional annotation of deduced proteins and differential abundance analysis of the corresponding transcripts were conducted on this non-redundant sequence subset.

Tentative descriptions and putative functions were assigned to deduced protein sequences by inference according to similarity with sequences in public databases. According to blastp similarity searches, only approximately two-thirds (20,859 of 32,884 queries, or 63.4 %) of the non-redundant set of deduced proteins returned significant hits (e-value < 1e-5) in the NCBI non-redundant protein sequence database. Similarly, searches of the UniProtKB-TrEMBL database returned 20,823 hits (63.3 % of query sequences at e-value < 1e-5), while the manually curated Swiss-Prot database returned 16,944 hits (51.5 %) using the same e-value cutoff. This indicates that, like other arthropods for which genome-wide sequence data has been generated [53], a large proportion of the *P. australiensis* transcriptome could not be annotated based on similarity to other proteins with known function.

**Fig. 2:**
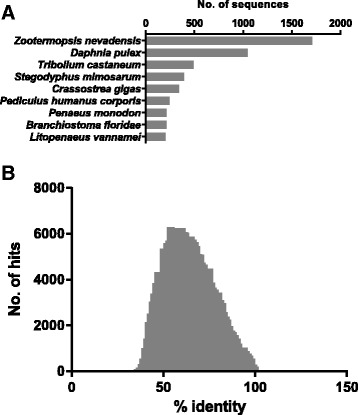
Annotation statistics for deduced proteins derived from transcripts expressed above an arbitrary lower abundance limit of 100 reads from any single library. **a** Source species for the best hit obtained for each query, limited to species with at least 200 hits. **b** Percent sequence similarity distribution for all blast hits

Gene ontology functions were assigned to a subset of sequences expressed at a minimum read coverage of 100 reads from any single library. Annotation statistics are presented in Fig. 2. The greatest number of hits from any single species (Fig. 2a) was obtained from dampwood termite (*Zootemopsis nevadensis*). This was also the case for a recent transcriptomic analysis of banana shrimp (*Fenneropenaeus merguiensis*) [54], and indicates that decapods are currently underrepresented in the available annotated sequence databases. A snapshot of overall sequence similarity with database proteins is presented in Fig. 2b, with the percentage identity for most hits falling between 50 and 80 percent.

### Differential transcript abundance

**Fig. 3:**
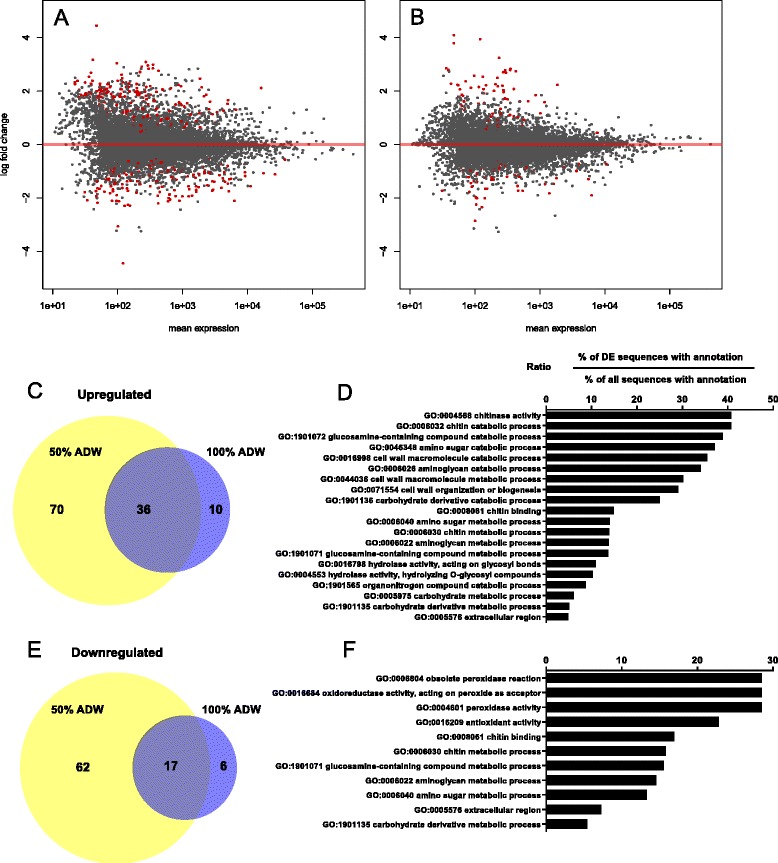
An overview of differential expression (DE) of transcripts in response to **a** 50 % acid drainage water (ADW) or **b** 100 % ADW according to transcript abundance estimates based on RNA-Seq read coverage. Data represent mean fold-change (log_2_ scale) relative to the control group, with mean transcript abundance (read coverage) on the x-axis. Transcripts with significant differential abundance relative to controls (*P*_adj_ < 0.05) are shown in red. **c** Venn diagram showing the number of transcripts with significantly higher differential abundance and biologically significant fold-difference (>2-fold) relative to control. **d** Bar chart showing significant enrichment of Gene Ontology (GO) functional terms (Fisher’s exact test *FDR* < 0.05) in the lists of significantly upregulated transcripts. Panels **e** and **f** represent the downregulated transcripts. Details of the transcripts in each GO category along with FDR values are available in Additional file *3*

The abundance of non-redundant protein-coding transcripts was estimated for each read library with the aid of software that utilises probabilistic models to resolve multiple read mappings across similar targets such as multi-isoform transcripts or those derived from closely related gene families [44, 55]. Differential abundance analysis based on these abundance estimates was determined using a generalised linear model (GLM)-based approach [45]. Transcripts exhibiting differential abundance relative to the control group with adjusted *P*-values (*P*_*adj*_) of less than 0.05 and relative abundances of 2-fold greater than or less than controls were deemed statistically significant and biologically relevant responses. Differential abundance analysis results are presented in Additional file 3. In response to 50 % ADW, 106 transcripts were significantly upregulated by at least 2-fold relative to controls and 79 transcripts were significantly downregulated. Exposure to 100 % ADW resulted in the significant upregulation of 46 transcripts and downregulation of 23 transcripts. Because the majority of transcripts exhibiting significant differential abundance in response to 100 % ADW were also differentially abundant in the 50 % ADW treatment group (Fig. 3c and e), we combined transcripts from both treatment groups for functional enrichment analysis. In either treatment group, 116 transcripts were significantly (*P*_*adj*_ < 0.05) up-regulated by at least 2-fold relative to the control group, and 85 transcripts were significantly (*P*_*adj*_ < 0.05) downregulated by at least 2-fold.

The overall magnitude of changes in transcript abundance relative to the control group was greater in the group exposed to 50 % ADW than in the 100 % ADW group (Fig. 3a, b, d and f). While this was somewhat unexpected, it may be attributable to differences in the bioavailability of dissolved metals due to differences in physicochemical factors such as pH or hardness. A previous study investigating metal speciation and bioavailability in ADW and diluent water from the same region has shown that metals present in ADW can precipitate after dilution to form fine particulates [56], which may be subsequently ingested by benthic feeders such as shrimp. However, the short exposure timeframe used in the present study probably rules out ingestion as a valid exposure route, and the greater overall response at the lower concentration is more likely to be related to changes in the speciation of dissolved metals.

Of the differentially expressed transcripts, only a small proportion could be annotated by whole-sequence similarity searches (Table 2) – 80 % of downregulated transcripts and 77.6 % of upregulated transcripts could not be annotated by sequence similarity, compared with 56.9 % of the whole dataset expressed above the minimum defined threshold. The proportion of differentially expressed transcripts that could not be annotated by any means 54.1 % and 57.8 % for down-regulated and up-regulated transcripts, respectively) was also markedly higher than for the whole dataset (34.7 %), making function inference of differentially expressed transcripts a challenge in the current study.

**Table 2:** Gene Ontology annotation statistics for deduced proteins corresponding to all transcripts above a minimum abundance threshold and differentially expressed subsets of these transcripts

	All transcripts with read coverage of > 100 in any library	Downregulated >2-fold in either 50 % or 100 % ASDW	Upregulated >2-fold in either 50 % or 100 % ASDW
Total number of deduced protein sequences	14,607	85	116
Sequences with annotations^a^	6293	17	26
Sequences that could not be annotated^a^	8314 (56.9 %)	68 (80 %)	90 (77.6 %)
Sequences with any functional inference^b^	9544	39	49
Sequences with no GO annotation	5063 (34.7 %)	46 (54.1 %)	67 (57.8 %)

Analysis was limited to transcripts with abundance in terms of raw read coverage of 100 reads per transcript from any single library

^a^According to BLAST2GO analysis, based on BLAST results only

^b^According to BLAST2GO analysis, based on BLAST results, domain homology searches using InterProScan, and ANNEX results

#### Functional enrichment analysis

**Fig. 4:**
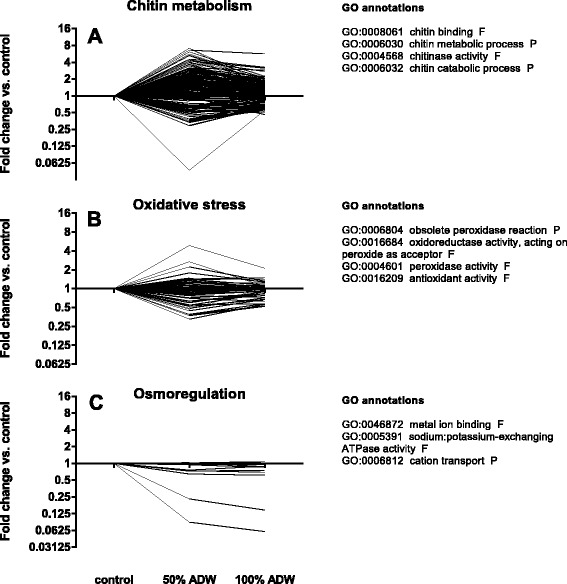
Relative abundance profiles for transcripts involved in environmental response pathways with relevance to exposure to acid drain water. **a** Transcripts involved in chitin biosynthesis or degradation **b** Transcripts involved in osmoregulation. **c** Transcripts associated with oxidative stress responses

In the lists of differentially expressed transcripts to which GO annotations could be assigned, terms that were significantly over-represented (Fisher’s exact test *FDR* < 0.05) could be placed into two broad functional categories – cuticle biosynthesis and oxidative stress (see Figs. 3d and f, and 4a and b). Details of the transcripts in each GO category along with FDR values are available in Additional file 4. These are discussed further in specific sections below. Although GO terms associated with osmoregulation (e.g. GO:0019829: ‘cation-transporting ATPase activity’) were not among the significantly enriched term lists, two transcripts encoding putative ion channels were significantly downregulated in response to ADW, and there was a general trend towards downregulation of transcripts to which such terms were assigned (Fig. 4c). Selected transcripts with putative functions related to environmental stress responses are listed in Table 3.

**Table 3:** Transcripts with functional annotations related to environmental stress responses exhibiting significant (*P*_adj_ < 0.05) and biologically relevant (log_2_ fold-change relative to controls > 1 or < -1) changes in abundance in either treatment group

Category and transcript ID	Assigned description	Gene Ontology terms (or conserved domains)	Log_2_FC 50 % ADW	P_adj_ 50 % ADW	Log_2_FC 100 % ADW	P_adj_ 100 % ADW
Oxidative stress responses / oxidoreductase activity						
*TR14472|c0_g2_i1|m.3443*	chorion peroxidase	F:peroxidase activity; F:heme binding; P:response to oxidative stress; P:peroxidase reaction	2.29	0.011	1.09	0.770
*TR84113|c0_g1_i2|m.82050*	aldehyde oxidase	F:electron carrier activity; F:flavin adenine dinucleotide binding; F:iron ion binding; F:2 iron, 2 sulfur cluster binding; F:oxidoreductase activity, acting on CH-OH group of donors; P:electron transport	1.02	0.0006068	1.16	5.41E-05
*TR21531|c3_g1_i2|m.10028*	peroxisomal n -acetyl-spermine spermidine oxidase-like	F:oxidoreductase activity	3.00	4.18E-05	2.84	0.00026
*TR29581|c1_g2_i2|m.19009*	spermatogenesis-associated protein 18-like protein	P:cellular response to stress; C:mitochondrial outer membrane	1.06	0.00024	0.86	0.011
*TR39455|c0_g1_i3|m.30019*	failed axon connections homolog	Thioredoxin-like domain PF13410 (PFAM)	4.46	5.82E-15	4.10	3.02E-12
*TR41138|c2_g1_i2|m.32139*	glutathione peroxidase 3	P:response to oxidative stress; F:glutathione peroxidase activity; P:glutathione metabolic process; P:peroxidase reaction	−1.17	0.012	−0.571	0.74
*TR41138|c2_g1_i1|m.32138*	glutathione peroxidase 6	P:response to lipid hydroperoxide; C:extracellular space; P:hydrogen peroxide catabolic process; C:extracellular vesicular exosome; F:selenium binding; F:transcription factor binding; F:glutathione peroxidase activity; P:protein homotetramerization; C:transcription factor complex; P:glutathione metabolic process; P:peroxidase reaction	−1.04	0.024	−0.476	0.86
*Metal binding activity*						
*TR70803|c0_g1_i1|m.66800*	metal transporter cnnm4	F:adenyl nucleotide binding	−0.38	0.52	−0.88	0.022
*Ion channel activity*						
*TR82036|c1_g1_i1|m.79405*	epithelial chloride channel partial	-	1.04	0.027	1.05	0.043
*TR64328|c0_g1_i1|m.60446*	na + k + -atpase alpha subunit	F:metal ion binding; P:ATP biosynthetic process; F:ATP binding; F:sodium:potassium-exchanging ATPase activity; C:sodium:potassium-exchanging ATPase complex	−1.38	0.21	−1.98	0.045
*TR61105|c5_g1_i2|m.55789*	na + k + -atpase alpha subunit	F:metal ion binding; P:ATP biosynthetic process; F:ATP binding; F:sodium:potassium-exchanging ATPase activity; C:sodium:potassium-exchanging ATPase complex	−0.46	0.291	−0.73	0.043
*Cell death / apoptosis*						
*TR80082|c0_g1_i2|m.75465*	programmed cell death protein 2	F:binding; P:programmed cell death; C:nucleus; C:cytoplasm	1.31	0.0814986	1.62	0.027945

*Abbreviations*: *Log*_*2*_ *FC* logarithm to base 2 of fold-changes in transcript abundance relative to controls, *ADW* acid drainage water, *P*_*adj*_, Benjamini-Hochberg corrected P value

#### Validation of selected changes in transcript abundance using qRT-PCR

Transcripts were selected for validation based on the magnitude of differential abundance, the existence of annotations relevant to the predicted responses, and overall transcript abundance. Two highly up-regulated transcripts (failed axon connections homologue [FACH] and cub serine protease [CSP]) and two highly down-regulated transcripts (Na+/K+ ATPase [NKA] and insulin-like growth factor binding protein 4 [IGP-BP4]) were selected based on magnitude of change regardless of the overall abundance. Additional transcripts (peroxisomal *n*-acetyl spermine oxidase [PAOX], chitooligosaccharidolytic beta-*N*-acetylglucosaminidase [CBAG], peptidyl-prolyl *cis-trans* isomerase a-like [PPI] and apolipoprotein d [APOD]) were selected based on relatively high overall abundance as well as significant differential abundance in either treatment group according to RNA-Seq.

**Fig. 5:**
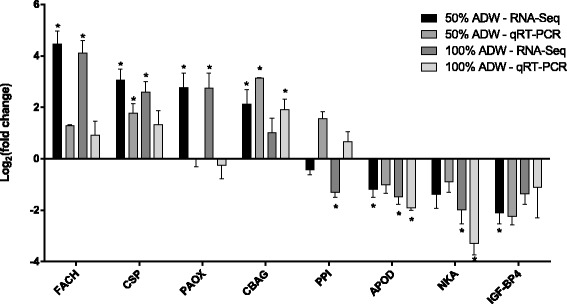
Validation of selected changes in transcript abundance using qRT-PCR. Data shown are log_2_-transformed mean abundance ratios in response to 50 % or 100 % acid drainage water relative to the control condition (river water). Asterisks (*) denote *P*_adj_ < 0.05 according to Benjamini-Hochberg multiple testing correction for the RNA-Seq data or one-way ANOVA followed by Dunnet’s multiple testing correction for qRT-PCR data. Abbreviations: FACH, failed axon connections homologue; CSP, cub serine protease; PAOX, peroxisomal n-acetyl spermine oxidase; CBAG, chitooligosaccharidolytic beta-N-acetylglucosaminidase; PPI, peptidyl-prolyl cis-trans isomerase a-like; APOD, apolipoprotein D; NKA, Na+/K+ ATPase; IGP-BP4,insulin-like growth factor binding protein 4

Relative abundances determined for selected transcripts using qRT-PCR exhibited variable consistency with values derived from RNA-Seq read coverage quantification (Fig. 5). Although fewer relative abundance values determined using qRT-PCR were statistically significant, and the magnitude of change generally lower, the direction of change for most transcripts (with the exception of PAOX and PPI) was consistent with the differential abundance values determined using RNA-Seq.

#### Osmoregulatory responses

The most well-studied osmoregulatory ion channel in euryhaline animals is the Na^+^/K^+^ ATPase (NKA), an active sodium and potassium pump that maintains haemolymph ionic strength under low-salinity conditions (for a review of osmoregulatory mechanisms in crustaceans, see [57]). In the gills of crustaceans, increased levels of NKA protein or overall Na^+^/K^+^ ATPase activity have been observed after transfer to hyposaline conditions (e.g. [58–60], which is consistent with the proposed central role in compensatory adaptation to low-salinity environments in order to maintain internal osmolarity. It follows that a decrease in the expression of ion pumps upon transfer from lower to higher salinity could be expected upon transfer of freshwater-adapted crustaceans from low to high salinity. A number of studies have confirmed that downregulation of NKA can indeed occur in decapod crustaceans after low-to-high salinity transfer [61, 62].

In the present study, transcripts encoding ion channels exhibited a general trend of reduced abundance in after exposure to ADW (Fig. 4c). A transcript encoding a putative NKA (TR64328|c0_g1_i1|m.60446) was one of the most strongly downregulated in the present study, with abundance decreasing by a factor of almost 4 relative to controls after exposure to 100 % ADW (Table 3; Fig. 4). The observed changes in mRNA level for this transcript were validated using qRT-PCR, which confirmed the significant downregulation of these transcripts similar magnitude (Fig. 5). Another putative NKA (TR61105|c5_g1_i2|m.55789) was also downregulated according to RNA-Seq data, but to a lesser extent (1.65-fold, *P*_*adj*_ = 0.045 in response to 100 % ADW). These responses are consistent with that which could be expected after a shift from osmoregulation in fresh water, which requires a high level of ion channel activity, towards osmoconformation after transfer to a moderately saline environment.

The electrical conductivity measured in sampled ADW was approximately 36.5 mS/cm, equivalent to a salinity of approximately 23‰, which is approximately two-thirds that of seawater. Factors such as a wide distribution range covering habitats with variable salinity, considerable phylogenetic diversity [36] and morphological variability [63] indicate that *P. australiensis* is a cryptic species complex, with sub-populations likely to display variable osmoregulatory adaptability. The present study shows that compensatory changes in mRNA levels encoding ion channels occurs rapidly (within 24 h) in response to moderate salinity levels. Among the transcripts annotated as NKAs, the two significantly down-regulated forms may represent suitable candidates for studying osmoregulatory responses in this species. A better understanding of the how these transcripts are regulated would help to predict the capacity of different subpopulations to survive increasing salinity, for example in coastal regions susceptible to the effects of sea level rise or in inland surface water systems affected by increasing salinity as a result of human activites.

#### Oxidative stress responses

There was a significant enrichment (Fisher’s exact test *FDR* < 0.05) of GO terms related to oxidative stress amongst the group of transcripts that were down-regulated in either treatment group (2-fold downregulation; *P*_*adj*_ < 0.05; Fig. 3c). This was somewhat surprising given the elevated metal concentrations in ADW, which could be expected to result in increased abundance of transcripts involved in oxidative stress [64]. Although abundance profiles for all oxidative stress-related transcripts showed up-regulation of some transcripts and down-regulation of others (Fig. 4b), the number of down-regulated transcripts in this functional category was clearly greater than the number of up-regulated transcripts.

#### Metabolic processes related to cuticle biosynthesis

The main structural component of the crustacean exoskeleton (or integument) is the cuticle, which is composed of complex polysaccharides such as chitin and glycosaminoglycan (GAG), proteins, lipoproteins and lipids. Numerous functional terms related to chitin and polysaccharide metabolism were significantly enriched (Fisher’s exact test *FDR* < 0.05) amongst both the upregulated transcripts (> 2-fold in either treatment group, *P*_*adj*_ < 0.05) and the downregulated transcripts (< 0.5-fold in either treatment group, *P*_*adj*_ < 0.05) (Fig. 3c; see also Fig. 4a). Functional terms included those related to chitin metabolism such as ‘chitin binding’, ‘chitin metabolic process’, ‘chitin catabolic process’ and ‘chitinase activity’, and others that were related to polysaccharide metabolism such as ‘carbohydrate metabolic process’, ‘cell wall macromolecule catabolic process’, ‘hydrolase activity, hydrolysing O-glycosyl compounds’ and ‘hydrolase activity, acting on glycosyl bonds’. Amongst the upregulated transcripts, almost all of the enriched functional terms were associated with chitin, carbohydrate or amino sugar metabolism. In the downregulated cohort, the proportion of terms associated with chitin, carbohydrate or amino sugar metabolism was less, however at least half of the terms were linked with these functions. Taken together, the differential abundance of a numerous of transcripts associated with cuticle formation may indicate a response to detrimental effects on cuticle structure and integrity caused by exposure to low pH conditions in the ADW.

The effects of extreme changes in pH have not been well studied in crustaceans. Exposure of shrimp to a very minor lowering of pH (pH 8 to pH 7.5) results in changes in the mineralisation status in the cuticle such as altered calcium:magnesium ratio, but no effects on cuticle thickness [65]. Exposure to water at pH 4 caused significant decreases in GAG and calcium levels in in the cuticle of intermolt crab in comparison to controls incubated at pH 7.5, but no significant differences in chitin content were observed [19]. Dissolution of GAG and calcium under acidic conditions has obvious implications for cuticle structure, which depends on mineralised calcium and magnesium for hardness and strength. In the present study, the extremely low pH values of the diluted (pH 3) and undiluted (pH 2.7) ADW would likely have caused demineralisation of the cuticle, possibly triggering metabolic responses related to biosynthesis of cuticle components.

Copper and zinc have been shown to cause a significant decrease in the abundance of chitinase mRNAs in the water flea *Daphnia magna* [66, 67]. Changes in chitinase mRNA levels have also been observed in response to copper exposure in the amphipod *Melita plumulosa* – the magnitude and direction of change differed with exposure route and concentration, with low dissolved copper (7 μg/L) resulting in elevated mRNA levels for a number of chitinases, while decreased levels were observed in response to high dissolved copper concentrations (43 μg/L) and particulate copper ingested through the diet [68]. Clearly, exposure to metals can result in the modulation of chitinase mRNA levels, the direction of which can vary according to concentration level. However, the biological reason of these changes are not currently known. It is possible that metal exposure could either inhibit or trigger moulting processes depending on the exposure concentration, and the relative abundance measured may also depend on the developmental stage of the animals studied. Since chitinase expression is required for moulting [69], the significant increase in the abundance of transcripts encoding a number of putative chitinases (e.g. TR30122|c0_g1_i1|m.19482 and TR51159|c0_g1_i1|m.44571) and other enzymes potentially involved in cuticle formation (e.g. TR46470|c0_g1_i3|m.38126 and TR73075|c1_g1_i1|m.68771, encoding putative chitooligosaccharidolytic beta-n-acetylglucosaminidases) observed in the present study indicates that moulting or cuticle repair processes may have been triggered by a combination of low pH and exposure to dissolved metals.

### Identification and annotation of additional transcripts belonging to gene families involved in environmental responses

*P. australiensis* has become an important ecotoxicology test organism for studying the challenges faced by aquatic biota as land use in the unique Australian landscape continues to change. There is a need to develop additional toxicological research tools for this species, such as rapid tests to detect changing levels of biomarkers related to exposure to specific toxicants, or studying the potency of environmental chemicals such as pesticides against specific targets such nuclear hormone receptors. To aid in these development of these tools, we identified transcripts related to selected environmental response pathways and undertook basic sequence analyse as described below. We believe providing this additional information about transcripts involved in key response pathways is important because computational tools used to predict protein coding regions necessarily omit short sequences such as those that encode metallothioneins and ignore selenocysteine codons (which are opal stop codons repurposed via a selenocysteine insertion sequence in the 3’ UTR), and because we would like to help to address the general paucity of rigorous functional annotations for crustacean protein sequences.

#### Metallothioneins

Metallothioneins (MTs) are small, cysteine-rich proteins that bind and transport essential trace metals [70, 71], participate in oxidative stress responses [72] and detoxify heavy metals by sequestration [73]. MTs are inducible by exposure to metal ions in solution, with some forms exhibiting specificity towards particular metals or groups of metals. Elevated MT transcript levels are widely considered as a good biomarker of metal exposure, despite wide variability in metal responsiveness among different the phyla and species studied (reviewed in [74]). The induction of MT gene expression, however, is not limited to metals or oxidative stress. For example, MT expression in white shrimp (*Litopenaeus vannamei*) can be induced by various stimuli such as salinity stress [75] and hypoxia [76]. Thus, MTs can be considered important for organismal responses to a range of environmental stressors and are hence highly relevant to the present study.

**Fig. 6:**
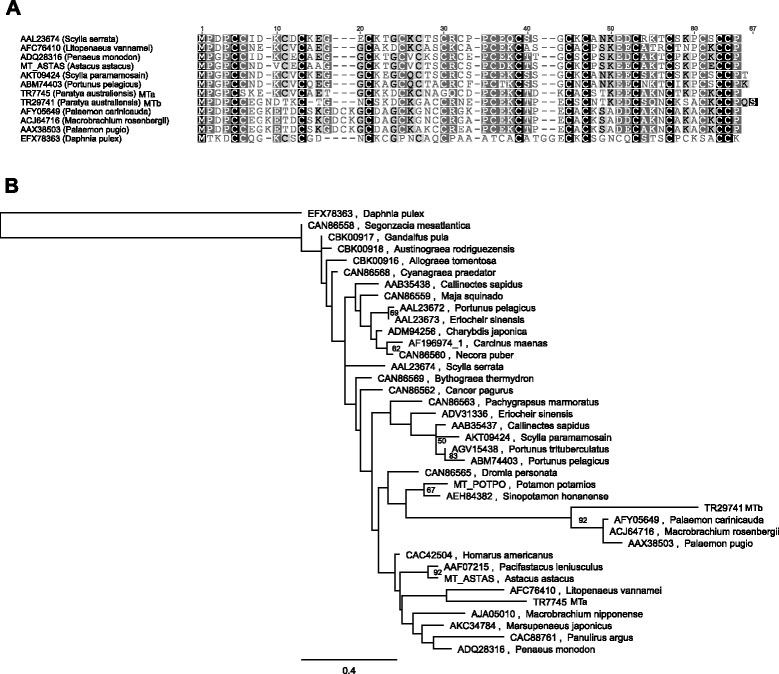
Sequence analysis of two putative metallothioneins identified in the P. australiensis transcriptome. **a** Multiple sequence alignments of deduced amino acid sequences from P. australiensis transcripts (denoted TR7745 and TR29741) with representative crustacean metallothioneins. Shading indicates similarity (unfilled, less than 60 % similarity; light grey, 60-80 %; dark grey, 80-99 %; black, 100 % similarity) **b** A maximum likelihood phylogenetic tree showing inferred relationships of P. australiensis MTa (encoded by TR7745) and MTb (TR29741)

Candidate ORFs of less than 300 nucleotides (corresponding to 100 amino acids) were omitted by the software used in the present study, necessitating manual similarity searches and functional annotation of putative metallothioneins. To identify MT-encoding transcripts in the *P. australiensis* transcriptome, similarity searches (blastn) were conducted against the entire set of assembled transcripts using MT cDNA sequences from giant river prawn (*Macrobrachium rosenbergii*; GenBank accession EU871044; [77]) and signal crayfish (*Pacifastacus leniusculus*; GenBank accession AF199482) as query sequences. This yielded two putative MT-coding sequences, TR7745|c0_g1_i1 and TR29741|c0_g1_i1. TR7745|c0_g1_i1 contains an ORF of 177 bp, encoding a putative MT of 58 amino acids (designated herein as MTa) with high similarity to a relatively large number of available MTs from decapods including shrimp such as *Litopenaeus vannamei* and *Macrobrachium nipponense*. TR29741|c0_g1_i1 contains a slightly longer ORF of 183 bp, encoding a deduced protein of 60 amino acids (designated MTb) bearing high similarity to MT sequences from the shrimp *Macrobrachium rosenbergii, Palaemon carinicauda* and *Palaemon pugio. P. australiensis* MTb appears to belong to a less well represented group of MTs previously described by Mahmood et al. [77], which contain 17 Cys residues instead of the 18 present in the majority of described decapod MTs. A multiple sequence alignment of representative crustacean MTs is provided in Fig. 6a.

According to a phylogenetic analysis (Fig. 6b), *P. australiensis* MTb, along with the crustacean 17-Cys variants, clearly form a clade separate from the majority of decapod MTs. It is not currently clear whether this is due to the fact that most researchers exploit publicly available sequence data to target new MTs for characterisation, thus generating a disproportionate number of annotated sequence data one isoform relative to others, or that the second form may be part of a recently diverged class of MT specific to a particular taxonomic group. Interestingly, *P. australiensis* MTb contains only 16 Cys residues and lacks the ‘DCKG’ motif identified by Mahmood et al. (2009) as a defining characteristic of the *M rosenbergii* and *Palaemon* MTs, resulting in the clear separation (with 92 % boostrap support) of the *P. australiensis* sequence from the remaining representatives in the clade.

**Fig. 7:**
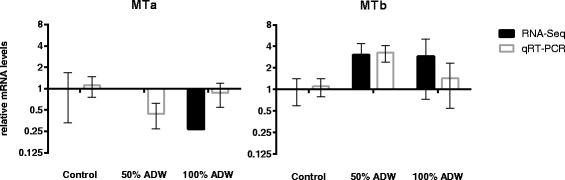
Changes in metallothionein expression in response to 50 % and 100 % ADW. No significant differences compared to the control condition were observed using either method (at *P*_*adj*_ < 0.05)

Analysis of changes in MT transcript abundance in response to ADW was determined using qRT-PCR (Fig. 7). The lack of significant changes in mRNA levels for MTa and MTb in the present study, despite the presence of elevated levels of heavy metals (e.g. Co, Ni, and Zn among others) in the exposure water, may be due to a number of factors. It is possible that the concentration of toxic metal ions in both 50 % and 100 % ADW were excessively high, resulting in general toxicity and the induction of responses other than compensatory mechanisms involving metal sequestration by MTs. Alternatively, the exposure time may not have been within a suitable range that induction could be observed, since the strong induction of MT gene expression can result in auto-regulation over time [78], and MT mRNA levels have been shown to vary rapidly within 24 h of exposure to metals [77]. Regardless of the lack of significant changes in the present study, the identification of two putative MT coding sequences in *P. australiensis* and the development of specific qRT-PCR assays for these transcripts, will allow more detailed follow-up studies on the responses to metal exposure in this species.

#### Glutathione peroxidases

Glutathione peroxidases (GPx) reduce hydroperoxides at the expense of glutathione, playing an important role in maintaining cellular redox balance and protecting against oxidative damage. In mammals, up to 8 GPx subfamilies have been described based on varying substrate specificity or biological function [79–81]. Mammalian GPx1-4 and GPx6 are classical selenoproteins; the 3’-untranslated region (3’-UTR) of their mRNA contains selenocysteine (Sec) insertion sequence (SECIS) elements that direct the read-through of an internal opal stop codon (TGA) and the addition of Sec to the nascent polypeptide chain. Although the specific functions have not been well studied, arthropods are known to express Sec-containing GPx proteins, most of which diverge somewhat from the mammalian subfamilies [79]. A recent phylogenetic analysis suggests that arthropod GPx homologues characterised to date form a clade with mammalian GPx3 [82], an extracellular form with broad substrate specificity [83].

In the present study, deduced proteins from six ORFs were identified as possible *P. austrliensis* GPx homologues after computational annotation of the non-redundant dataset. By performing manual ORF searches of the corresponding full-length transcripts, it was evident that some of these sequences represented truncated forms due to the automated misinterpretation of putative Sec-encoding opal stop codons. With the aid of the Selenoprotein Prediction Server [84], four putative full-length GPx candidates were identified. By comparison of the coding sequences it appears that two of these putative GPx sequences (TR41138|c2_g1_i1|m.32138 and TR41138|c2_g1_i2|m.32139) are likely to represent splice variants derived from the same gene, while the other two (TR21053|c0_g1_i1|m.9601 and TR12356|c0_g1_i1|m.1706) are clearly from different genes but may be close paralogues. The remaining two transcripts annotated as GPx candidates represented partial coding sequences and are not discussed further here. Both TR41138|c2_g1_i1|m.32138 and TR41138|c2_g1_i2|m.32139 exhibited a significant (*P*_*adj*_ < 0.05) and likely biologically relevant (fold-change < 0.5) decrease in abundance in response to 50 % ADW (Table 3).

A phylogenetic analysis of the full-length *P. australiensis* GPx sequences (Additional file 5) showed that all four were more similar to the vertebrate GPx3 subfamily than to other vertebrate GPxs. However, according to our analysis there is a clear separation of the vertebrate and arthropod clades and indication that multiple subtypes of arthropod GPx may exist. Determining the function of these subtypes, particularly their substrate specificity, would help to delineate subclasses within the arthropod glutathione peroxidases.

#### Nuclear hormone receptors

In arthropods, nuclear receptors (NRs) regulate fundamental developmental processes such as organ development, sexual differentiation and moulting. Arthropod NRs are fewer in number and less divergent than NRs found in vertebrates, particularly amongst the NR3 subfamily which has only a single representative in arthropods compared to 9 in mammals and in up to 15 in fish [85, 86]. Despite this, some crustacean NRs have been shown to be vulnerable to modulation by xenobiotics. Two examples are HR96, which can be activated or inhibited by various pesticides, steroids and fatty acids [87], and ecdysone receptor (EcR), a moulting hormone receptor targeted by certain classes of insecticides that can have potent off-target effects in aquatic crustaceans [88].

We identified putative NRs in the *P. australiensis* transcriptome by selecting deduced proteins containing both a conserved ligand-binding and a conserved DNA-binding domain with similarity to known NRs. To assign tentative sub-family classifications to putative NRs, a phylogenetic tree was constructed based on multiple sequence alignment of the DNA binding and ligand binding domains (Additional file 6). This approach is valid because, despite a complex evolutionary history, it has been shown that all NRs shared a single common ancestor [86]. At least one putative homologue was present in the *P. australiensis* transcriptome for all but one NR (knirps-like nuclear receptor) annotated in the two most well-studied arthropod genomes (*Drospohila melanogaster* and *Daphnia pulex*).

## Conclusions

The high proportion of arthropod proteins with unknown function represents a significant challenge for researchers in the field of environmental toxicogenomics, which relies on similarity-based functional inference to decipher gene regulatory networks related to toxicant effects. In the present study, most of the transcripts exhibiting differential abundance after exposure to ADW could not be annotated due to the lack of homologous sequences with known function. The relative availability of genome-wide sequence data for decapod crustaceans is low compared with other arthropods such as insects. Efforts in this area will undoubtedly be aided by the i5K project, which aims to sequence the genomes of 5000 arthropods including a list of 20 decapods [89], and transcriptomics surveys such as the present study will assist researchers studying species for which genome sequencing projects are unlikely to be available in the near future. The annotated transcriptome data presented herein represents a valuable resource not only for ecotoxicology researchers working with *P. australiensis*, but also for those studying the effects multiple stressors in decapod crustaceans and other invertebrates. Increased effort towards identifying the function highly conserved orthologous protein families that occur throughout the arthropoda, or indeed lineage-specific variants, would help to better understand the molecular processes that occur in response to complex environmental stressors. Recent work indicates that under certain conditions altered geochemistry in acid sulfate soil-affected areas can result in the prolonged acidification of drains in reclaimed agricultural land [18] and in groundwater beneath affected lake margins [90] years after rainfall water levels return to normal patterns. The resilience of resident populations of aquatic crustaceans to long-term, low-level increases in the levels of acidity, salinity and dissolved metals in such environment may rely on environmental response pathways such as those identified in the present study. Further analysis of the deregulation of transcripts and pathways involved in these responses will aid in the development of biomarkers for metal exposure, salinity and pH stress.

## Additional files

Additional file 1: Table S1. Primer sequences used for qRT-PCR; **Table S2**: Physicochemical water quality parameters for acid drainage water and control river water; **Table S3**: Basic physicochemical water quality parameters for undiluted ADW, diluted ADW and river water during the laboratory exposures; and **Table S4**: Paired-end read counts obtained for each sample.) (DOCX 16 kb)
Additional file 2: Phylogenetic analysis of all publicly available sequence data representing *P. australiensis* COI variants. (PDF 602 kb)
Additional file 3: Differential abundance analysis results, functional annotations and putative descriptions of transcripts passing the minimum abundance threshold. (XLSX 2717 kb)
Additional file 4: Functional enrichment analysis results including p-values and false discovery rates. (XLSX 36 kb)
Additional file 5: Phylogenetic analysis of glutathione peroxidase sequences. (PDF 591 kb)
Additional file 6: Phylogenetic analysis of nuclear hormone receptor sequences. (PDF 793 kb)

## Abbreviations

ADW: Acid drainage water
APOD: Apolipoprotein d
BUSCO: Benchmarking universal single-copy orthologue
CBAG: Chitooligosaccharidolytic beta-*N*-acetylglucosaminidase
CD: Conserved domain
cDNA: complementary DNA
COI: Cytochrome c oxidase subunit I
CSP: Cub serine protease
EC: Electrical conductivity
EcR: Ecdysone receptor
FACH: Failed axon connections homologue
FDR: False discovery rate
GAG: Glycosaminoglycan
GLM: Generalised linear model
GO: Gene ontology
GPx: Glutathione peroxidase
IGF-BP4: Insulin-like growth factor binding protein 4
Log_2_FC: Logarithm to base 2 of fold-change
MT: Metallothionein
NKA: Na+/K+ ATPase
NR: Non-redundant
NR: Nuclear receptor
ORF: Open reading frame
PAOX: Peroxisomal *n*-acetyl spermine oxidase
PPI: Peptidyl-prolyl *cis-trans* isomerase a-like
qPCR: quantitative PCR
qRT-PCR: quantitative reverse-transcriptase PCR
RNA-Seq: High-throughput sequencing of messenger RNA
Sec: Selenocysteine
SECIS: Selenocysteine insertion sequence
UTR: Untranslated region
