# Supplementary material for: De novo assembly and analysis of changes in the protein-coding transcriptome of the freshwater shrimp Paratya australiensis (Decapoda: Atyidae) in response to acid sulfate drainage water
Source: BMC Genomics. 2016 Nov 7;17:890. doi: 10.1186/s12864-016-3208-y (PMC5100079; doi:10.1186/s12864-016-3208-y)
Supplement: Additional file 1: Table S1. — Primer sequences used for qRT-PCR; Table S2: Physicochemical water quality parameters for acid drainage water and control river water; Table S3: Basic physicochemical water quality parameters for undiluted ADW, diluted ADW and river water during the laboratory exposures; and Table S4: Paired-end read counts obtained for each sample.) (DOCX 16 kb) [file 12864_2016_3208_MOESM1_ESM.docx]

Table S1. Oligonucleotide primers used for quantitative reverse-transcriptase PCR

| Abbreviation | Description (inferred by sequence similarity) | Target transcript\|CDS ID | Sense primer 5'-3' | Antisense primer 5'-3' | Amplicon length (bp) |
| --- | --- | --- | --- | --- | --- |
| PAOX | Peroxisomal n-acetyl spermine oxidase | TR21531\|c3_g1_i8\|m.10032 | GGAACCGACTCTTGGAATGA | ACTGGGGTCGACAGTTTCAC | 152 |
| eIF2A-K | eIF-2a kinase | TR70866\|c0_g1_i1\|m.67038 | GCGGCCTTATCTAGCAACTG | TCAATTGGCCATTGTTTTCA | 182 |
| IGF-BP4 | Insulin-like growth factor-binding protein 4-like | TR59573\|c0_g1_i1\|m.54179 | TGTTAGCTGGCAGTGTGTCC | ACGCCTTTGGTAGTTGATGG | 150 |
| FACH | Failed axon connections homologue | TR39455\|c0_g1_i3\|m.30019 | GCTGACTGTGCTGTTTTCGG | TTGCAATAATCGCCGAGGGA | 110 |
| MTa | Metallothionein a | TR7745\|c0_g1_i1 | TTCGTCTTCTTGCTGAACCA | GAAAAGTGTGTTTGCGCTGA | 197 |
| MTb | Metallothionein b | TR29741\|c0_g1_i2 | ATTTCCAACATGCCTGATCC | CGGATTTGCAATTCTGTGAG | 166 |
| RPS7 | 40s ribosomal protein s7 | TR81695\|c0_g1_i1\|m.78571 | AAAAGAAATTCAGCGGCAAA | CAGACGCACCCTGATTCTTT | 194 |
| CBAG | Chitooligosaccharidolytic beta-N-acetylglucosaminidase | TR73075\|c1_g1_i1\|m.68771 | GAGCAACGCTTTCTCCAAAC | CTCCACTGATCTGGGTTGGT | 201 |
| CSP | af357226_1cub-serine protease | TR32624\|c0_g1_i1\|m.21567 | GGAGGAAACCACCCAAAAAT | TGCTCTGTTGTCTTCCATCG | 250 |
| APOD | apolipoprotein d | TR10051\|c0_g1_i1\|m.1336 | CTTCAGCACAGGCAAAATGA | TGTTGCAGAACGAACCATGT | 165 |
| PPI | peptidyl-prolyl cis-trans isomerase a-like | TR39567\|c2_g2_i1\|m.30370 | CTTGCCAAAAATGGGTTTGT | AAGCCTCCAACTGCTTTTGA | 189 |
| NAKA | Na+/K+ ATPase, alpha subunit | TR64328\|c0_g1_i1\|m.60446 | TCCAACACACGGAAATCGTA | ATACCCATGGCAACACCAAT | 160 |

Table S2. Water quality parameters for acid drainage water and river water used for laboratory exposures in the present study

|  | Control river water | Acid drainage water |
| --- | --- | --- |
| ***Physico-chemical parameters*** |  |  |
| pH | 7.87 | 3.17 |
| EC (µS/cm) | 211 | 36,548 |
| Alkalinity (meq/L) | 1.52 | - |
| Acidity | - | 6.411 |
| Chloride (meq/L) | 32 | 12,000 |
| Sulfate (mg/L) | 10 | 4,100 |
| Sodium (mg/L) | 18.6 | 5,320 |
| Potassium (mg/L) | 3.94 | 119 |
| Calcium (mg/L) | 8.16 | 1,090 |
| Magnesium (mg/L) | 4.9 | 1,040 |
| NH_4_-N (mg/L) | < 0.005 | 4.0 |
| NO_2_-N (mg/L) | < 0.005 | < 0.005 |
| Total nitrogen (mg/L) | 1 | 5 |
| Total phosphorus (mg/L) | 0.007 | 0.015 |
| DOC | 7 | 16 |
| ***Metals*** |  |  |
| Al (mg/L) | 165 | 1,400 |
| Ag (µg/L) | < 0.02 | < 0.1 |
| As (µg/L) | 47 | 465 |
| Cd (µg/L) | < 0.06 | 1.5 |
| Co (µg/L) | 0.20 | 569.8 |
| Cr (µg/L) | < 0.4 | < 2 |
| Cu (µg/L) | 2.1 | 4 |
| Ni (µg/L) | 1.4 | 490 |
| Pb (µg/L) | 0.40 | 7.2 |
| Zn (µg/L) | 47 | 465 |
| Fe (mg/L) | 0.228 | 55.8 |
| Mn (mg/L) | < 0.05 | 13 |

Abbreviations: EC, electrical conductivity; NH4-N, nitrogen as ammonium; NO2-N, nitrogen as nitrites.

Table S3. Water quality parameters during laboratory exposures

| Condition | pH | DO (mg/L) | Conductivity (mS/cm) |
| --- | --- | --- | --- |
| Control | 7.55 | 5.57 | 0.290 |
| 50% ADW | 4.17 | 5.07 | 20.2 |
| 100% ADW | 3.05 | 5.04 | 38.7 |

Table S4. Paired read yields from Illumina 100-cycle paired-end mRNA sequencing

| Sample | Number of paired reads |
| --- | --- |
| Control A | 22,071,697 |
| Control B | 21,011,640 |
| Control C | 24,518,416 |
| 50% ADW A | 21,549,284 |
| 50% ADW B | 16,246,991 |
| 50% ADW C | 22,620,833 |
| 100% ADW A | 22,893,480 |
| 100% ADW B | 21,284,052 |
| 100% ADW C | 21,366,486 |
