# Supplementary material for: De novo assembly and analysis of changes in the protein-coding transcriptome of the freshwater shrimp Paratya australiensis (Decapoda: Atyidae) in response to acid sulfate drainage water
Source: BMC Genomics. 2016 Nov 7;17:890. doi: 10.1186/s12864-016-3208-y (PMC5100079; doi:10.1186/s12864-016-3208-y)
Supplement: Additional file 2: — Phylogenetic analysis of all publicly available sequence data representing P. australiensis COI variants. (PDF 602 kb) [file 12864_2016_3208_MOESM2_ESM.pdf]

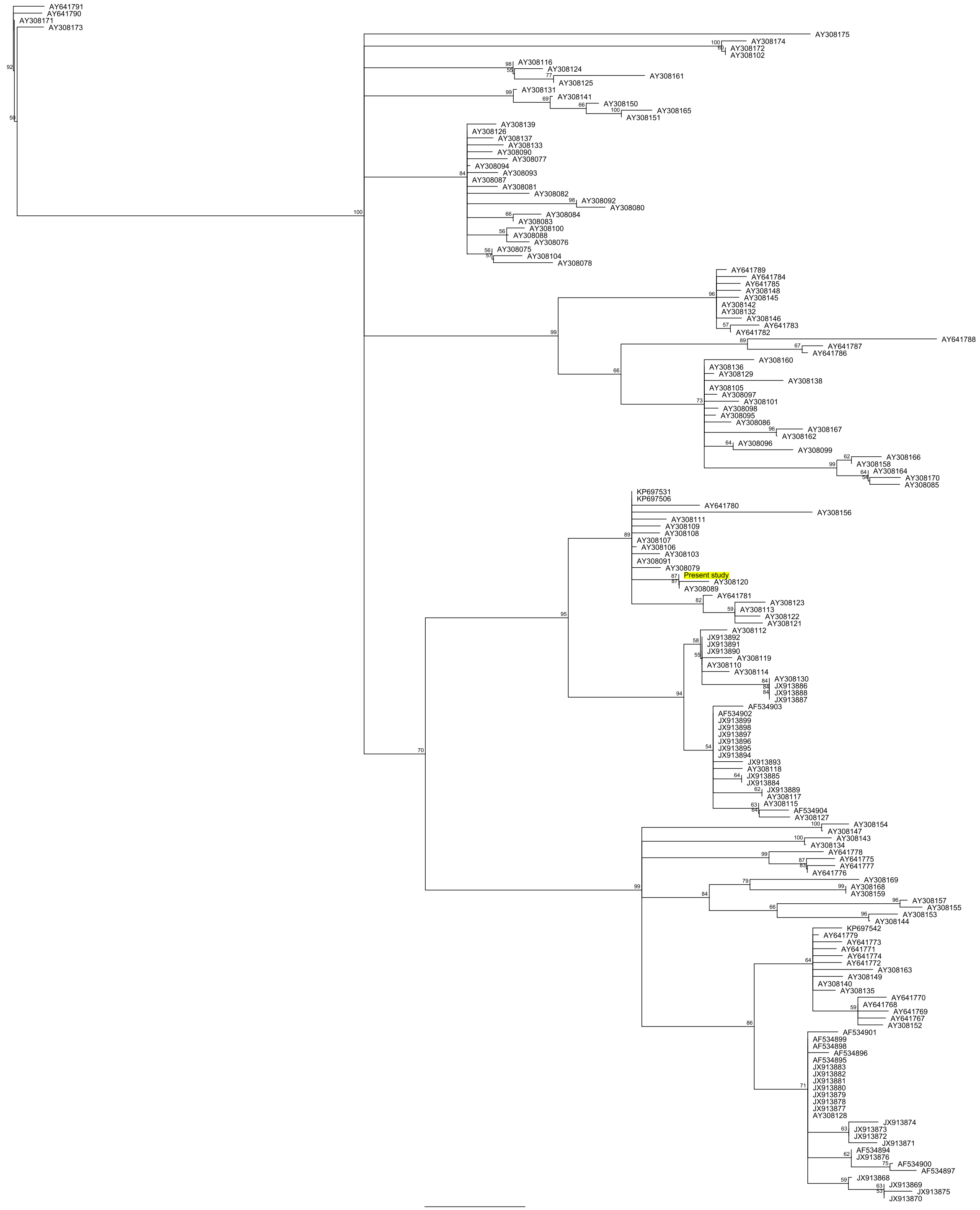

Supplementary figure 1. Neighbour-joining phylogenetic tree based on partial mitochondrial cytochrome c oxidase sequences showing the relatedness of the P. australiensis strain used in the present study to isolates reported by Cook et al. (2006).
