# Supplementary material for: De novo assembly and analysis of changes in the protein-coding transcriptome of the freshwater shrimp Paratya australiensis (Decapoda: Atyidae) in response to acid sulfate drainage water
Source: BMC Genomics. 2016 Nov 7;17:890. doi: 10.1186/s12864-016-3208-y (PMC5100079; doi:10.1186/s12864-016-3208-y)
Supplement: Additional file 5: — Phylogenetic analysis of glutathione peroxidase sequences. (PDF 591 kb) [file 12864_2016_3208_MOESM5_ESM.pdf]

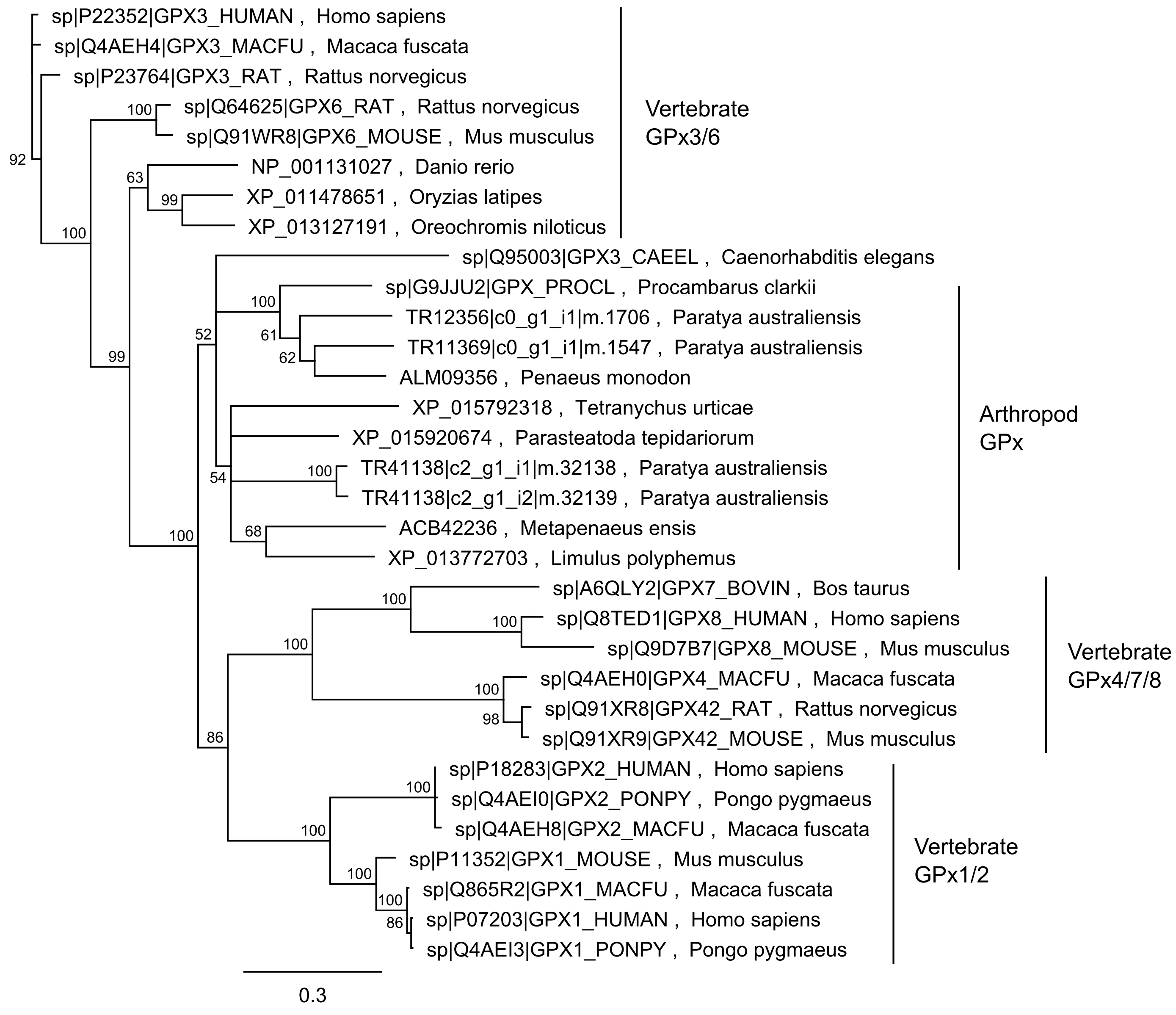

Phylogenetic tree of deduced amino acid sequences showing inferred evolutionary relationships among selected glutathione peroxidases (GPx) from crustaceans and other arthropods in comparison with vertebrate GPx subfamilies. Putative GPx sequences identified in the *P.australiensis* transcriptome are labelled with Trinity transcript identifiers and the open reading frame identifiers from Transdecoder.
