# Supplementary material for: De novo assembly and analysis of changes in the protein-coding transcriptome of the freshwater shrimp Paratya australiensis (Decapoda: Atyidae) in response to acid sulfate drainage water
Source: BMC Genomics. 2016 Nov 7;17:890. doi: 10.1186/s12864-016-3208-y (PMC5100079; doi:10.1186/s12864-016-3208-y)
Supplement: Additional file 6: — Phylogenetic analysis of nuclear hormone receptor sequences. (PDF 793 kb) [file 12864_2016_3208_MOESM6_ESM.pdf]

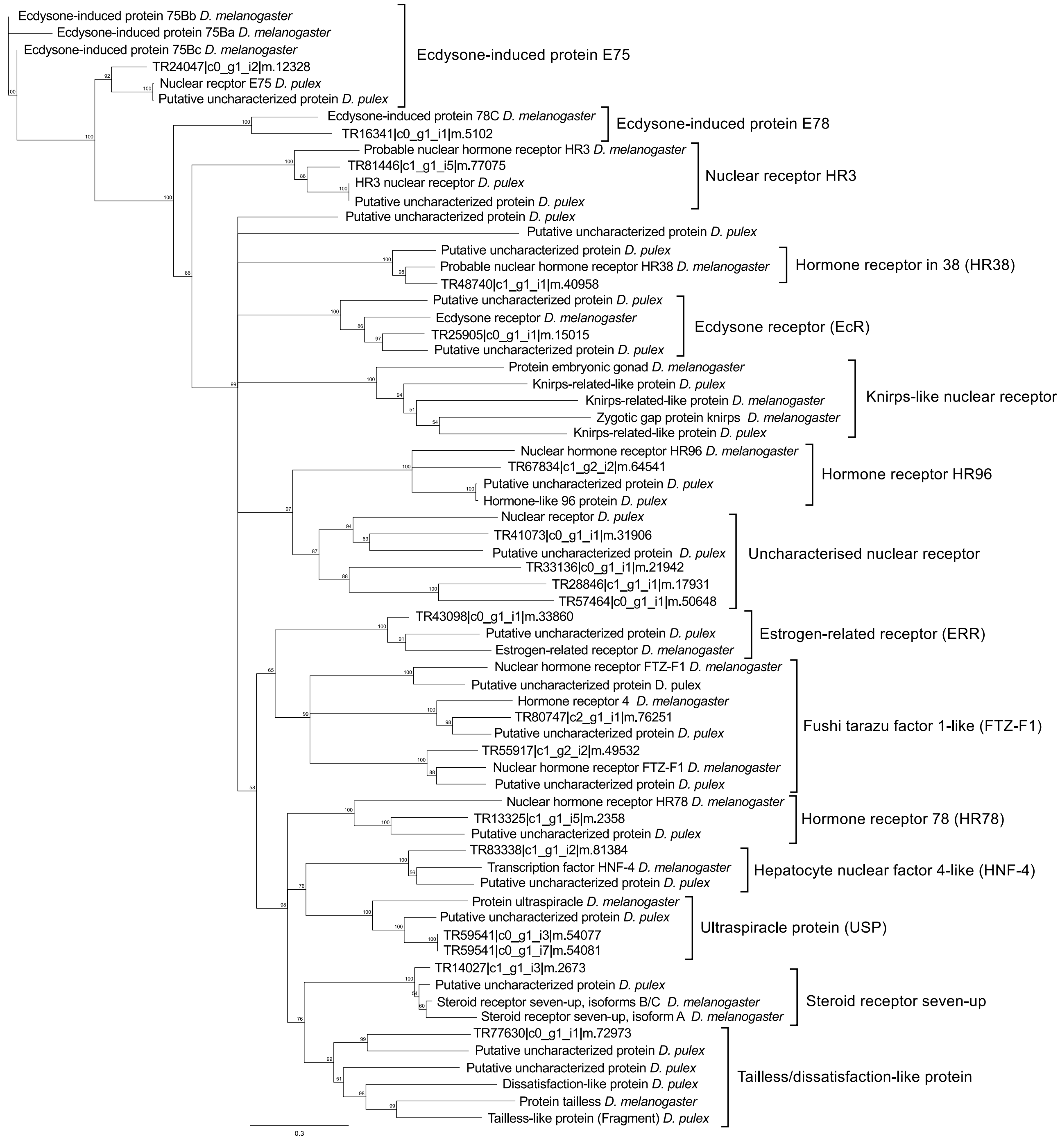

Additional file 6. A neighbour-joining phylogenetic tree showing tentative placement of canditate nuclear receptors identified in the *Paratya australiensis* transcriptome within known invertebrate nuclear receptor subfamilies. *P. australiensis* sequences are labelled with Trinity transcript identifiers (TR*xxxxx*|cx_g*x*_i*x*) and Transdecoder coding sequence identifiers (m.*xxxxx*).
